# Prostate imaging features on magnetic resonance imaging of young patients

**DOI:** 10.31744/einstein_journal/2022AO0024

**Published:** 2022-11-18

**Authors:** Renan Kinoshita Suguino, Thaís Caldara Mussi, Fernando Morbeck Almeida Coelho, Ronaldo Hueb Baroni

**Affiliations:** 1 Hospital Israelita Albert Einstein São Paulo SP Brazil Hospital Israelita Albert Einstein, São Paulo, SP, Brazil.

**Keywords:** Prostate, Prostatic neoplasms, Magnetic resonance imaging

## Abstract

**Objective:**

To identify magnetic resonance imaging findings of the prostate in young adults, including symptomatic and asymptomatic patients. The aim of this study is to evaluate the main aspects of prostate imaging in young patients.

**Methods:**

A total of 102 patients under 40 years of age, who underwent prostate magnetic resonance imaging between January 2016 and January 2019, were included in this study. The patients were divided into two groups: symptomatic for prostatitis (Group 1) and asymptomatic (Group 2). Magnetic resonance imaging scans were anonymized and interpreted by a radiologist blinded for clinical information. The study evaluated peripheral zone signal in T2-weighted sequences, diffusion and apparent diffusion coefficient map; peripheral zone enhancement pattern; seminal vesicles and periprostatic fat.

**Results:**

All evaluated criteria did not present statistically significant differences between the two groups. The most common pattern was heterogeneous hyposignal on T2 (57.9% in Group 1 and 57.8% in Group 2; p=0.506), mild diffuse / wedge-shaped areas of hypointensity on apparent diffusion coefficient map (61.4% in Group 1 and 64.4% in Group 2; p=0.931) and early post-contrast enhancement (73.7% in Group 1 and 68.9% in Group 2, p=0719).

**Conclusion:**

The magnetic resonance imaging aspect of young patients showed no differences between symptomatic and asymptomatic patients.

## INTRODUCTION

Prostate cancer (PCa) is the most frequent neoplasm in males, and approximately 190 thousand new cases and 33 thousand deaths were estimated for 2020, in the United States.^(1)^ In addition, the incidence of PCa is directly related to age, increasing in the patients aged over 50 years. The prevalence in men aged >65 years is approximately 60%.^(2)^

Currently, there is an exponential rise in magnetic resonance imaging (MRI) examinations of the prostate, notably in the context of PCa.^(3,4)^ The most common indications are detection of clinically significant PCa (csPCa), guiding target biopsies, staging, active surveillance, and assessment of biochemical recurrence.^(5,6)^

Therefore, most prostate MRI studies are performed in older patients, and few studies are conducted to evaluate the prostate in young patients, including normal appearance and abnormal findings.^(7–10)^ Indeed, neither of the studies mentioned investigated the MRI prostate findings of young men, either symptomatic or asymptomatic.

## OBJECTIVE

To identify magnetic resonance imaging findings of the prostate in young adults, including symptomatic and asymptomatic patients.

## METHODS

This is a single-center retrospective study, approved by the Institutional Review Board (IRB) of *Hospital Israelita Albert Einstein* (HIAE), with a waiver for informed consent (CAAE: 27353019.7.0000.0071; #4.084.225). We searched our database from January 2016 to January 2019 for patients aged 40 years or less, who had been submitted to prostate MRI.

### Patient population

A total of 102 patients were found and none of them were excluded. Patients included in this study were divided into two groups. The asymptomatic group (Group 1) was composed of patients with conditions such as infertility, alteration in sperm count, evaluation of prostate cysts and epididymis, hematospermia or detection of clinically significant neoplasia. The symptomatic group (Group 2) comprised patients with clinical suspicion of prostatitis.

The patients of both groups had a median age of 35.29 years (range 18-40 years), a mean serum prostate-specific antigen (PSA) of 2.40ng/mL (range 0.12-36.76ng/mL).

### Magnetic resonance imaging protocol

All patients underwent MRI on 3T scanners with a pelvic phased-array coil and no endorectal coil. A routine protocol was used, including high-resolution sequences, diffusion-weighted imaging (DWI), apparent diffusion coefficient (ADC) map, and T1-weighted (T1W) post-contrast sequences, as showed in table 1. Extracellular gadolinium-based contrast media (Magnevist, Bayer, Leverkusen, Germany) was injected at a dose of 0.2cc/kg and a rate of 2cc/sec.

**Table 1:** Magnetic resonance imaging parameters

	T2w axial	T2w sagittal	T2w coronal	DWI	DCE
Sequence type	TSE	TSE	TSE	DWI	GRE
FOV (cm)	16 x 16	19 x 19	40 x 40	22 x 22	22 x 22
Matrix size	320 x 320	256 x 256	384 x 384	128 x 128	288 x 288
TR (msec)	5200	4000	1200	4000	3.5
TE (msec)	141	146	143	57	1.37
Fat suppression	-	-	-	SPAIR	Fat saturation
EPI factor	-	-	-	128	-
Acceleration factor	2	2	2	2	2
Signal averages	3	3	1.4	6-10	1
Section thickness (mm)/gap	3/0	3/0	1/0	3/0	3/0
*b* factor (mm^2^/sec)	-	-	-	50, 400, 800 and 1500	-
Pixel bandwidth (Hz)	203	300	723	2056	720

T2w: T2-weighted imaging; DWI: diffusion weighted imaging; DCE: dynamic contrast enhanced; TSE: turbo spin echo; GRE: gradient-echo; FOV: field of view; TR: time of repetition; TE: time of echo; EPI: echo-planar factor; msec: millisecond; sec: second.

### Imaging analysis

All sequences were anonymized by one of the authors. Imaging interpretation was performed on a Picture Archiving and Communication System (PACS) workstation (KODAK/Carestream; Carestream Health, Rochester, NY).

A radiologist with 3-year-experience in abdominal radiology, and with more than 750 studies read, reviewed the prostate MRI, blinded for clinical history and original reports.

The peripheral zone was evaluated for its T2W signal, DWI and ADC map and dynamic contrast-enhanced (DCE) pattern. For the T2W imaging, the radiologist should classify the peripheral zone as diffusely hypointense (when hypointensity covered more than 80% of volume of the peripheral zone), heterogeneously hypointense (when between 30 and 80%) and hyperintense (when less than 30% of peripheral zone was hypointense).

Regarding ADC map and DWI, the radiologist should classify the peripheral zone as normal; mild diffusely hypointense or hypointense with wedge-shaped pattern on the ADC map; moderate or marked homogeneous hypointensity; moderate or severe heterogeneous hypointensity.

The pattern of DCE was classified as mild and progressive enhancement; diffuse and early enhancement; heterogeneous and early enhancement.

In addition, seminal vesicle walls and their contents, and periprostatic fat were also evaluated. Seminal vesicle walls were classified as normal or thickened, and seminal vesicle content was characterized as normal or associated with blood, hyperproteic component or calculi. Periprostatic fat was categorized into normal or associated with inflammatory changes.

### Statistical analysis

Variables were compared between the two groups using the Mann-Whitney U test for numerical values, and the *χ*^2^ test or Fisher’s exact test for categorical variables. A value of p<0.05 was considered statistically significant. Statistical analysis was performed using the software SPSS for Windows, v22.0, IBM.

## RESULTS

As showed in table 2, there was no significant difference between symptomatic and asymptomatic groups in evaluation of peripheral zone on T2W imaging, DWI, and ADC map.

**Table 2:** Features on prostate magnetic resonance imaging in asymptomatic (Group 1) and symptomatic (Group 2) patients

Feature		Group 1 n (%)	Group 2 n (%)	p value
Peripheral zone T2W signal				0.506
	Diffuse hypointensity	15 (26.3)	15 (33.3)	
	Heterogeneous hypointensity	33 (57.9)	26 (57.8)	
	Hyperintensity	9 (15.8)	4 (8.9)	
Percentage of the peripheral zone surface with hypointensity				0.376
	<30%	14 (28.0)	7 (17.1)	
	30% to 80%	11 (22.0)	8 (19.5)	
	>80%	25 (50.0)	26 (63.4)	
DWI and ADC map				0.931^#^
	Normal	17 (29.8)	11 (24.4)	
	Mild diffuse hypointensity/wedge-shaped hypointensity	35 (61.4)	29 (64.4)	
	Moderate or marked homogeneous hypointensity	1 (1.8)	1 (2.2)	
	Moderate or severe heterogeneous hypointensity	4 (7.0)	4 (8.9)	
DCE pattern				0.719^#^
	Without contrast	3 (5.3)	2 (4.4)	
	Mild and progressive enhancement	12 (21.1)	12 (26.7)	
	Diffuse and early enhancement	25 (43.9)	15 (33.3)	
	Early heterogeneous enhancement	17 (29.8)	16 (35.6)	
Seminal vesicle walls				0.281
	Normal	49 (86.0)	35 (77.8)	
	Thickened	8 (14.0)	10 (22.2)	
Seminal vesicle content				0.530*
	Normal	52 (91.2)	39 (86.7)	
	Hematic / hyperproteic / calculus	5 (8.8)	6 (13.3)	
Periprostatic fat				>0.999*
	Normal	51 (89.5)	40 (88.9)	
	Inflammatory changes	6 (10.5)	5 (11.1)	
Total		57 (100)	45 (100)	

χ^2^ test;

* Fisher’s exact test;

# likelihood ratio test.

T2W: T2-weighted imaging; DWI: diffusion weighted-imaging; ADC: apparent diffusion coefficient; DCE: dynamic contrast enhanced.

Heterogeneous low signal appeared in 57.9% of patients in both suspected and non-suspected prostatitis groups. High signal in the peripheral zone was observed in 15.8% of asymptomatic and 8.9% of symptomatic groups (p=0.506).

The distribution in the DWI and ADC map findings showed predominance of mild diffuse hypointensity/wedge-shaped hypointensity in ADC map, with a prevalence of 61.4% and 64.4% in asymptomatic and symptomatic groups, respectively (p=0.931).

**Figure 1:**
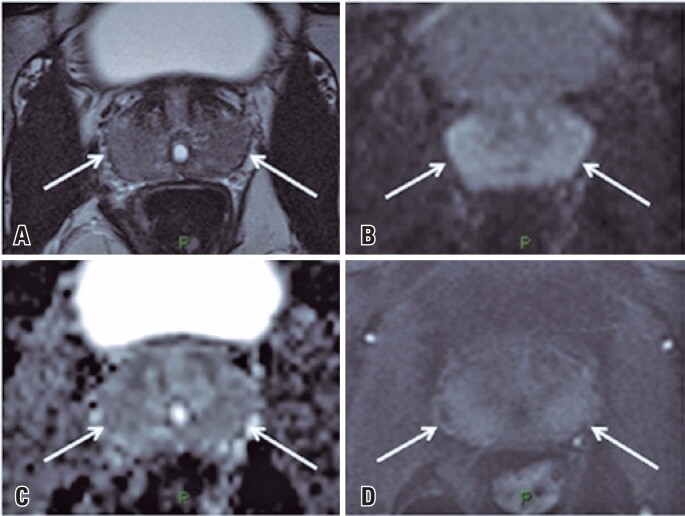
Prostate magnetic resonance imaging of a 31-year-old asymptomatic man to evaluate an epididymal cyst. The exam shows diffuse hyposintensity on T2W (arrows in A), with areas of diffusion restriction seen on DWI and ADC map (arrows in B and C) and early diffuse contrast enhancement (arrows in D)

**Figure 2:**
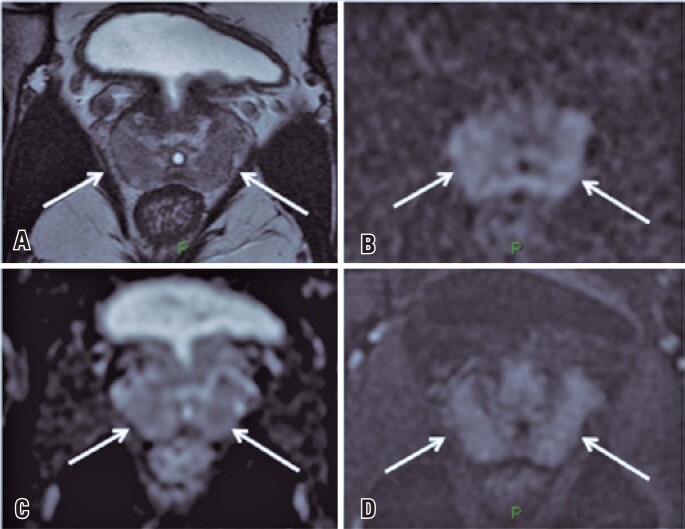
Prostate magnetic resonance imaging of a 39-year-old man with suspected acute prostatitis. The imaging study shows aspects that resemble those seen in figure 1 (in an asymptomatic patient): diffuse hypointensity on T2W (arrows in A), with areas of diffusion restriction seen on DWI and ADC map (arrows in B and C) and early diffuse contrast enhancement (arrows in D)

Moreover, there was no significant difference in the DCE pattern. The diffuse and early enhancement pattern was found in 43.9% of asymptomatic patients and 33.3% of symptomatic patients, while the mild and progressive enhancement pattern was found in 26.7% of symptomatic *versus* 21.1% in asymptomatic group (p=0.719) (Figures 1 and 2).

There was no significant difference in the seminal vesicle walls and their content. The seminal vesicle walls were thickened in 14.0% in group 1 and 22.2% in the symptomatic group, and had hematic, hyperproteic, calculus contents in 8.8% of asymptomatic patients and 13.3% of those with clinically suspected prostatitis (p=0.281 and p=0.530, respectively).

There was no significant difference in the evaluation of periprostatic fat, which presented inflammatory changes in 10.5% (Group 1) and 11.1% (Group 2) of MRI studies (p>0.999).

We also searched for patients who performed prostate biopsy in an interval of 12 months after MRI, trying to correlate imaging and pathological findings. No patients in the symptomatic group and only two patients in the asymptomatic group underwent prostate biopsy. One patient was 40 years old and submitted to MRI for suspected PCa and increased PSA levels; the biopsy was negative for malignancy and only showed mixed inflammatory changes (lymphomononuclear cells and neutrophils). The other patient was 39 years old, in active surveillance for PCa (ISUP 1) and underwent MRI that showed an indeterminate lesion (PI-RADS 3).Biopsy was performed almost one year later and showed small foci of sparse PCa (ISUP 2).

## DISCUSSION

For many years, prostate MRI was mainly performed for local staging of PCa; however, nowadays, it has a wide role in prostate management, including detection of clinically significant prostate neoplasm, planning target biopsies, radiation therapy planning, and follow-up of patients in active surveillance.^(11,12)^ Therefore, when dealing with young patients, the most common indications for prostate MRI are slightly different. In our series, almost half of patients submitted to prostate MRI were under investigation for acute prostatitis. The other half underwent examination due to various conditions, including detection of clinically significant neoplasm, investigation of infertility, hematospermia, leukospermia, cysts in epididymis, seminal vesicles or prostate.

In our study, the most common prostate findings were evaluated in young adults and compared between asymptomatic and symptomatic patients; there was wide variability in imaging features. The finding of homogeneous T2W hypointensity, classically described in peripheral zone of young and healthy patients^(10)^ was not the most prevalent in asymptomatic patients, an there was a predominance of heterogeneous low signal in this group. The minority of the patients presented a peripheral hyperintensity finding, classically described for older and healthy patients.^(10)^

The study also demonstrated a predominance of mild diffuse/wedge-shaped hypointensity on the ADC map. Medved et al.^(9)^ studied young patients (20-28 years) and evaluated the pattern of prostate MRI before, during and after ejaculation, observing a significant reduction in peripheral zone T2W signal after ejaculation, verified in the quantitative (visual) and quantitative (ROI measurement) analyses. In this same article, ADC map presented a significant signal drop only in quantitative evaluation.^(9)^ In our study, we did not assess patients’ sexual abstinence interval, which could be a confounding bias.

Acute prostatitis on MRI tends to have low signal on T2W imaging, associated with mild to moderate diffusion restriction due to more inflammatory cellular infiltrates. This condition may also increase perfusion, and the pattern is commonly band-like, wedge-shaped, or diffuse, rather than focal, round, oval, or irregular.^(13,14)^ In our study, clinically suspected patients for acute prostatitis demonstrated imaging findings similar to those found in asymptomatic patients. The low signal on T2W imaging described for acute prostatitis was seen in the majority of asymptomatic and symptomatic patients (over 80% of patients), with no significant difference. The features of mild to moderate diffusion restriction, which is described in cases of prostatitis,^(13)^ were also similar in symptomatic and asymptomatic groups. There was a higher prevalence of diffuse and early enhancement in symptomatic patients than in the asymptomatic group (43.9% *versus* 33.3%), an expected and described finding for patients with prostatitis.^(14)^Our study shows that those findings should not be independently considered for acute prostatitis since they can be routinely found in asymptomatic patients.

Other characteristics that suggest local inflammation, such as periprostatic fat edema and seminal vesicle wall thickening, had a similar incidence in asymptomatic and symptomatic groups; *i.e*., approximately 10% for periprostatic fat edema, and 14.0% (Group 1) *versus* 22.2% (Group 2) for thickened seminal vesicle walls. These findings could be explained by the fact a high number of young patients can present acute inflammatory findings and subclinical symptoms.

This study had some limitations. First, only one radiologist read the cases and there was no interobserver agreement to be evaluated. Second, quantitative imaging analysis was not evaluated. And finally, we did not correlate the imaging findings with prostate biopsy, since this was not the aim of our study and there were few histopathologic results, as expected in a scenario of younger patients.

## CONCLUSION

In conclusion, we observed the prostate of young men showed a wide variability of imaging findings, with similar characteristics between symptomatic and asymptomatic patients for suspected prostatitis.
